# DNA methylation analysis reveals distinct methylation signatures in pediatric germ cell tumors

**DOI:** 10.1186/1471-2407-13-313

**Published:** 2013-06-27

**Authors:** James F Amatruda, Julie A Ross, Brock Christensen, Nicholas J Fustino, Kenneth S Chen, Anthony J Hooten, Heather Nelson, Jacquelyn K Kuriger, Dinesh Rakheja, A Lindsay Frazier, Jenny N Poynter

**Affiliations:** 1 Department of Pediatrics, University of Texas Southwestern Medical Center, Dallas, TX 75390, USA; 2 Department of Molecular Biology, University of Texas Southwestern Medical Center, Dallas, TX 75390, USA; 3 Department of Pathology, University of Texas Southwestern Medical Center, Dallas, TX 75390, USA; 4 Center for Cancer and Blood Disorders, Children’s Medical Center, Dallas, TX 75390, USA; 5 Department of Pediatrics, Division of Pediatric Epidemiology and Clinical Research, Minneapolis, MN 55455, USA; 6 Masonic Cancer Center, University of Minnesota, Minneapolis, MN 55455, USA; 7 Division of Epidemiology and Community Health, University of Minnesota, Minneapolis, MN 55455, USA; 8 Department of Community and Family Medicine, Section of Biostatistics and Epidemiology, Dartmouth Medical School, Hanover, NH 03755, USA; 9 Dana Farber Cancer Institute, Boston 02115MA, USA

**Keywords:** Germ Cell Tumor, Teratoma, DNA Methylation, Imprinting

## Abstract

**Background:**

Aberrant DNA methylation is a prominent feature of many cancers, and may be especially relevant in germ cell tumors (GCTs) due to the extensive epigenetic reprogramming that occurs in the germ line during normal development.

**Methods:**

We used the Illumina GoldenGate Cancer Methylation Panel to compare DNA methylation in the three main histologic subtypes of pediatric GCTs (germinoma, teratoma and yolk sac tumor (YST); N = 51) and used recursively partitioned mixture models (RPMM) to test associations between methylation pattern and tumor and demographic characteristics. We identified genes and pathways that were differentially methylated using generalized linear models and Ingenuity Pathway Analysis. We also measured global DNA methylation at LINE1 elements and evaluated methylation at selected imprinted loci using pyrosequencing.

**Results:**

Methylation patterns differed by tumor histology, with 18/19 YSTs forming a distinct methylation class. Four pathways showed significant enrichment for YSTs, including a human embryonic stem cell pluripotency pathway. We identified 190 CpG loci with significant methylation differences in mature and immature teratomas (q < 0.05), including a number of CpGs in stem cell and pluripotency-related pathways. Both YST and germinoma showed significantly lower methylation at LINE1 elements compared with normal adjacent tissue while there was no difference between teratoma (mature and immature) and normal tissue. DNA methylation at imprinted loci differed significantly by tumor histology and location.

**Conclusion:**

Understanding methylation patterns may identify the developmental stage at which the GCT arose and the at-risk period when environmental exposures could be most harmful. Further, identification of relevant genetic pathways could lead to the development of new targets for therapy.

## Background

Aberrant DNA methylation has been implicated in the etiology of multiple types of cancer, and has the potential to be especially relevant in germ cell tumors (GCTs) due to extensive epigenetic reprogramming that occurs in the germ line and early embryo during normal development. Histologically, GCTs can be divided into germinomas and non-germinomas. Germinomas (GERs; also called seminomas in the testis and dysgerminomas in the ovary) are tumors of undifferentiated germ cells that retain markers of pluripotency. In contrast, non-germinomas undergo differentiation to resemble somatic-type tissues (teratomas) or extra-embryonic structures (yolk sac tumor (YST) and choriocarcinoma). Studies of testicular GCTs have suggested that global methylation patterns differentiate the main histologic subtypes, with seminomas exhibiting global DNA hypomethylation while nonseminomas exhibit higher levels of methylation [1-3]. Initially, these data supported a theory that the methylation status indicated the embryonic stage of development of the primordial germ cell (PGC) when the tumor arose, with seminomas arising from a hypomethylated PGC and nonseminomas originating following *de novo* methylation of PGCs [1]. However, the hypomethylation observed in IGCNU (Intratubular Germ Cell Neoplasia, Unspecified), which is believed to be the precursor of both seminomas and non-seminomas, would suggest that both seminomas and nonseminomas are derived from a hypomethylated PGC [2]. Importantly, these alterations in methylation may be clinically relevant as DNA methylation has been shown to predict response to cisplatin treatment in an adult testicular cancer cell line [4].

Few studies have evaluated DNA methylation in pediatric GCTs [5-9]. Of these, three have identified hypermethylation in the promoter of tumor suppressor genes [6-8] while two others have identified unique methylation patterns that can help distinguish between tumors of different histologic subtypes [5,9]. In addition, alterations in genomic imprinting, which is controlled by DNA methylation, have been identified in GCTs [10-12].

In adolescents, as in adults, GCTs can present as germinomas, non-germinomas or a mixture of the two types. Young children less than 5 years of age, in contrast, develop primarily yolk sac tumors and teratomas. While yolk sac tumors are malignant at any age, the significance and clinical management of teratomas remain controversial. Mature teratomas contain fully differentiated tissues, and when occurring in the testis of pre-pubertal males or in the ovary are benign tumors [13]. In contrast, immature teratomas are characterized histologically by the presence of immature tissues, especially neural tissue. Higher-grade immature teratomas (those containing a higher percentage of immature elements) are often considered malignant and treated with cytotoxic chemotherapy [14]. While studies have identified clinical [15] and radiographic [16,17] features that separate mature from immature teratomas, the molecular signature of immature teratomas is not well understood. To date, methylation patterns have not been compared in mature and immature teratomas in the pediatric age group.

Given the important role of epigenetic reprogramming in normal germ cell development, additional studies of DNA methylation are likely to increase our understanding of the etiology of pediatric GCTs. In this analysis, we evaluated differences in DNA methylation in cancer-related and imprinted genes by tumor and patient characteristics in a series of 51 pediatric GCTs, including YSTs, germinomas and teratomas (mature and immature). In addition, we evaluated global hypomethylation at LINE1 elements in a subset of the samples.

## Methods

### Study samples

GCTs from pediatric and adolescent patients (ages 0–21 years) were obtained from the Cooperative Human Tissue Network (Columbus, OH) and from Children’s Medical Center Dallas (CMC). Tumors were resected at initial diagnosis and snap frozen at -70°C. Pathology reports were also provided. Data were available for tumor histology, tumor location (gonadal or extragonadal), sex, and age at diagnosis. Normal adjacent tissue was also available for five of the tumors (four ovarian and one testicular) in our case series. Diagnosis was verified by a pediatric pathologist prior to molecular analysis and only samples with >70% tumor cellularity of pure histological subtypes were included.

This analysis used existing data with no personal identifiers; therefore, the study was deemed exempt from review by the Institutional Review Boards of the University of Minnesota and the University of Texas Southwestern Medical Center and CMC.

### DNA extraction and bisulfite conversion

Genomic DNA was isolated from GCT tissue and paired normal adjacent tissue (when available) using either the TRIzol^®^ extraction method (Invitrogen Life Technologies, California) or a QIAamp DNA Mini Kit (Qiagen Sciences, Maryland) according to the manufacturer’s recommended protocol. DNA yield was quantified using 1 μl DNA on a NanoDrop™ spectrophotometer (Thermo Scientific, Maryland). Extracted DNA was stored at -80°C until further analysis.

Prior to methylation analysis, 1 μg genomic DNA was treated with sodium bisulfite to convert unmethylated cytosines to uracil using the EZ DNA Methylation Kit (Zymo Research, Orange, CA) according to manufacturer’s protocol.

### GoldenGate cancer methylation panel

DNA methylation at 1505 CpG loci in 807 cancer-related genes was evaluated using the GoldenGate Cancer Methylation Panel I (Illumina, Inc.) in the Biomedical Genomics Center at the University of Minnesota following the manufacturer’s protocol as described [18]. Replicates were included, including four duplicates that were included on both arrays and five duplicates that were included within one array.

### Pyrosequencing

Array methylation results were validated by Pyrosequencing using a PyroMark MD80 Pyrosequencer (Qiagen) in a subset of the samples (N = 41 samples from CHTN). Five pyrosequencing assays were designed for regions targeting the CpG loci on the array that had significant methylation differences between yolk sac tumor and other histologic subtypes. Briefly, PCR primers and sequencing primers were designed using PSQ Assay Design software (Qiagen, Inc) to capture the array CpG and as many neighboring CpGs as possible. Methylation at imprinted loci was evaluated using assays described in Woodfine et al. [19]. Primers and conditions are available upon request. Global LINE1 methylation was measured by pyrosequencing 4 CpG loci in the LINE1 region as previously described [20]. LINE1 was measured in triplicate for each sample.

Commercially available Epitect methylated and unmethylated DNA standards were used as controls (Qiagen). In addition, a sequencing primer control and a no template control were included for each assay. The level of methylation for each CpG within the target region of analysis was quantified using the Pyro Q-CpG Software.

### Preparation of total RNA

Total RNA was prepared from fresh frozen tumor tissue. 30–50 mg of tissue was homogenized using Tissue Miser (Fisher Scientific, Pittsburgh, PA) in TRIzol^®^ Reagent (Invitrogen, Carlsbad, CA); approximately 1 mL TRIzol^®^ per 50 mg of tissue was used. After incubation for 30 minutes at room temperature, phase separation was done using chloroform (200 μL/1 mL Trizol^®^). Sample was shaken vigorously, centrifuged at 13000 rpm at 4°C, and aqueous phase removed. RNA precipitation was done using 70% ethanol. To remove contaminant genomic DNA, on-column DNase digestion was done using RNase-Free DNase Digestion Kit (Qiagen, Valencia, CA). RNA isolation was done per manufacturer’s instructions using RNeasy^®^ Mini Kit (Qiagen, Valencia, CA) and final elution performed in 20 μL H_2_O. Quantity and purity was assessed using NanoDrop™ 1000 spectrophotometer (Thermo Fisher Scientific, Wilmington, DE). Absorbance ratios at 260/280 nm and 260/230 nm were used to verify purity. Quality was further assessed by visualization of 28S and 18S bands after performing gel electrophoresis (1% agarose in 1X Tris-EDTA-Acetate Buffer).

### Quantitative RT-PCR

cDNAs were synthesized from 1 μg of purified RNA using RT^2^ First Strand Kit (SABiosciences, Frederick, MD). Real-time quantitative PCR gene expression profiling was performed using a Wnt pathway-specific array (SABiosciences, Frederick, MD). Arrays profiled 84 pathway-specific genes with validated primers and contained internal control primers to assess genomic DNA contamination, RNA quality, and PCR amplification efficacy. RT-qPCR was performed on Applied Biosystems 7500 Real-Time PCR System (Carlsbad, CA) using RT^2^ SYBR^®^ Green qPCR Master Mix (SABiosciences, Frederick, MD) as a fluorophore for amplicon detection. PCR conditions were as follows: 95°C × 10 minutes, 95°C for 15 seconds then 60°C for 1 minute × 40 cycles, followed by a dissociation stage per manufacturer’s protocol. Gene expression was normalized to endogenous HPRT, β-actin (ACTB) and glyceraldehyde-3-phosphate dehydrogenase (GAPDH), as these internal reference genes exhibited the least variation among the five internal reference genes evaluated. Fold change of gene expression was determined using the 2^(-ΔΔCt)^ method, and compared yolk sac tumors (n = 4) to germinomas (n = 3). We performed unsupervised hierarchical cluster analysis using web-based PCR data analysis software (http://www.sabiosciences.com/pcrarraydataanalysis.php). Raw gene expression data and calculations are shown in Additional file 1: Tables S2-S8, . Gene expression among histologic subtypes was compared using a type 3 *t*-test (Additional file 1: Table S7).

Real time quantitative RT-PCR for SOX2 and DNMT3B (N = 34 samples) was measured using a human embryonic stem cell PCR array (SA Biosciences). Fold change of gene expression was determined using the 2^(-ΔΔCt)^ method, and differences by tumor histology were measured using generalized linear models.

### Statistical analysis

To understand differences in methylation patterns by tumor histology, we evaluated the three main histologic subtypes as determined by pathology review (YSTs, dysgerminomas, and teratomas) using the analytic techniques described below.

### GoldenGate methylation data

Using the GoldenGate array, the methylation status of a CpG site is calculated as the variable β, which is the ratio of the fluorescent signal from the methylated allele to the sum of the fluorescent signals of both methylated and unmethylated alleles [18]. These values range from 0 (unmethylated) to 1 (fully methylated). GenomeStudio software (Illumina, Inc) was used to calculate the average methylation values (β) from the ~30 replicate methylation measurements for each CpG locus. We used raw average β values without normalization. GenomeStudio software was also used to assess data quality for each CpG loci. We omitted all CpG loci where ≥ 25% of the samples had a detection p-value > 0.05 (N = 16, 1%). X-linked CpG loci (N = 84) were also removed, resulting in 1,405 loci for analysis.

The remaining analyses for the array data were conducted in R [21]. Methylation differences were evaluated using unsupervised hierarchical clustering with the Manhattan metric and average linkage as previously described [22]. We used recursively partitioned mixture modeling (RPMM) to test associations between methylation status and tumor (histology and location) and demographic (age at diagnosis and sex) characteristics as described [23] and implemented [22,24]. Briefly, samples are assigned to a methylation class using a model-based form of unsupervised clustering. Permutation-based tests (with 10,000 permutations) were used to test for associations between methylation class and covariates: we used a chi-squared test for categorical covariates (tumor histology, tumor location, and sex), and a Kruskal-Wallis test statistic to test associations between methylation class and age.

We then used a series of generalized linear models (GLM) to identify genes that were differentially methylated in YSTs and teratomas as previously described [22]. We accounted for multiple testing by controlling the false-discovery rate (FDR) [25]. Q-values were computed using the q-value package in R.

Ingenuity Pathway Analysis (IPA; Ingenuity Systems) was used to identify pathways that were enriched in the list of CpG loci with significantly different methylation in YSTs compared with other histologic subtypes of tumors and in immature teratomas compared with mature teratomas. We implemented an IPA Core analysis with HUGO gene symbol as the identifier. For the analysis of YSTs, we restricted the analysis to CpG loci with up-regulated methylation (effect size > 1.0). For the comparison of mature and immature teratomas, we restricted the analysis to CpG loci with down-regulated methylation in immature teratomas. Both analyses included only CpG loci that were significant after controlling for multiple comparisons (q-value < 0.05)

### Pyrosequencing data

Analysis of pyrosequencing data was conducted using SAS v. 9.2 (SAS Institute, Cary, NC). For the array validation assays, Pearson correlation coefficients and p-values are reported for correlation between Pyrosequencing and GoldenGate data.

For the imprinted loci, we would expect methylation to be ~50%. We categorized samples into three groups: 1) <33% methylation (hypomethylated), 2) 33-66% methylation (median methylation), and 3) >66% methylation (hypermethylation) as previously described [11,26]. A Fisher’s exact test was used to evaluate statistical significance of any differences in methylation by tumor histology and location.

Global LINE1 measure was evaluated by calculating the mean methylation level across the 4 LINE1 CpG loci. The mean was then averaged across the three replicates for each sample. Differences in LINE1 methylation across tumor histology (YST, germinoma, mature teratoma, immature teratoma, normal adjacent), tumor location, sex and age group were evaluated using a GLM with LINE1 methylation as the outcome variable.

## Results

### Characteristics of the study samples

Tumor specimens from 51 cases of pediatric GCT ranging in age from 0 – 21 years were included in this analysis, including 19 yolk sac tumors (YSTs), 22 teratomas (8 immature and 14 mature), and 10 germinomas (Table 1). The YSTs were evenly distributed among boys and girls while the majority of cases with a germinoma or teratoma were female. Information on race/ethnicity was not available for the cases. Normal adjacent DNA was available for five cases (four ovary and 1 testis). Correlation coefficients for replicates were ≥ 0.95 for all samples. There were no significant differences in methylation values when we compared samples extracted by the Trizol method with samples extracted by QIAamp after adjustment for tumor histology (p > 0.05).

**Table 1:** Selected characteristics of the study samples

	**Yolk Sac Tumor**	**Immature Teratoma**	**Mature Teratoma**	**Germinoma**
	**N (%)**	**N (%)**	**N (%)**	**N (%)**
**Total**	19	8	14	10
**Age**				
**Median (range)**	1 (0 – 19)	5 (0 – 21)	4.5 (0 – 15)	12 (7 – 17)
**Sex**				
**Male**	10 (53)	1 (87)	4 (29)	0
**Female**	9 (47)	7 (12)	10 (71)	10 (100)
**Tumor location**				
**Ovary**	4 (21)	4 (50)	6 (43)	10 (100)
**Testis**	6 (32)	1 (12)	0	0
**Extragonadal**	9 (47)	3 (37)	8 (57)	0

### Methylation differences by tumor histology

**Figure 1:**
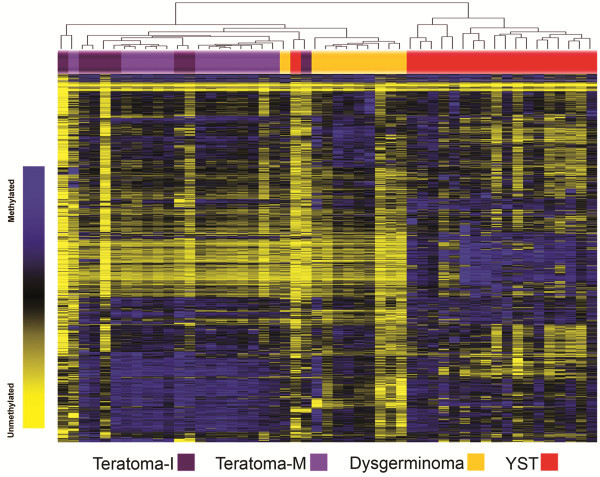
**Unsupervised hierarchical clustering of CpG methylation in GCTs by tumor histology.** Heat map from unsupervised hierarchical clustering based on Manhattan distance and average linkage of the 1404 autosomal CpG loci that passed initial quality control checks. Colored bars represent histologic subtype of the tumor. Light purple represents mature teratoma, dark purple represents immature teratoma, orange represents germinoma and red represents yolk sac tumor. Samples are in columns (N = 51) and CpG loci are in rows. Blue indicates high level of methylation (51-100%), black equals 50% methylation, and yellow indicates low level of methylation (0-49%).

**Figure 2:**
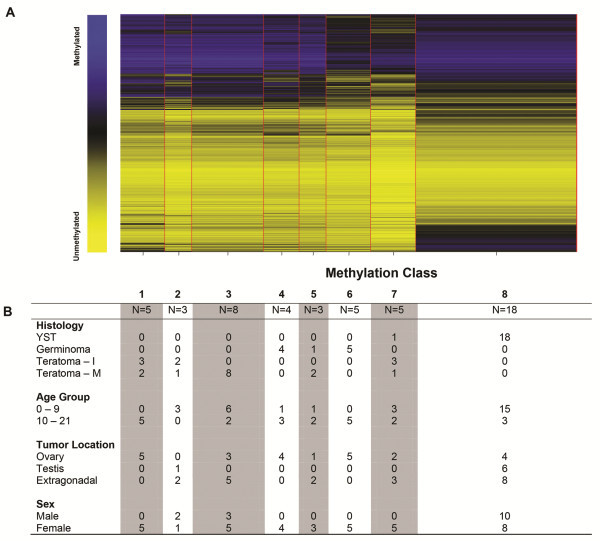
**Recursively partitioned mixture model (RPMM) of CpG methylation in GCTs. A.** Columns represent methylation class generated by RPMM and rows represent the average methylation within the class at each CpG site. Blue represents methylated and yellow represents unmethylated. The width of the row is proportional to the number of samples included in the methylation class. **B.** Characteristics of the tumors in each methylation class.

Unsupervised clustering of methylation data revealed differences by tumor histology (Figure 1). Modeling the methylation data with RPMM resulted in 8 methylation classes (Figure 2). Methylation classes were significantly associated with tumor histology (p < 0.0001): class 8 included 18/19 YSTs and classes 4–6 included all germinomas (Figure 1). Eight of the mature teratomas comprised their own methylation class (Class 3) while the remaining six were classified with either immature teratomas or dysgerminomas. Methylation class was also significantly associated with tumor location (p = 0.005), sex (p = 0.008) and age at diagnosis (p < 0.001).

In comparisons of YSTs with the other histologic types, we identified 703 CpG sites with statistically significant differences in methylation (q-value < 0.05). Of the 233 CpGs most significantly associated with YST histology (q-value < 2.2E-16), the majority (96%) had increased methylation. Twenty-three CpG loci with the most significant q values also had an adjusted fold change in β ≥ 2.75, indicating that YSTs had methylation levels ≥ 2.75 times higher than tumors of other histologic types at these loci (Table 2).

**Table 2:** Top 23 genes with differential methylation in YST

**CpG Locus**	**Effect size**^**a**^	**q-value**	**Δ beta YST1**^**b**^	**Δ beta YST2**^**b**^
***HLA*. *F *_ *E402 *_*F***	3.69	<2.2E-16	0.86	0.81
***WT1*_ *E32 *_*F***	3.41	<2.2E-16	0.86	0.83
***RASSF1*_ *E116 *_*F***	3.16	<2.2E-16	0.87	0.79
***CYP1B1*_ *E83 *_*R***	3.13	<2.2E-16	0.74	0.54
***CCNA1***_***E7***_***F***	3.13	<2.2E-16	0.67	0.78
***SLC22A3***_***E122***_***R***	3.12	<2.2E-16	0.82	0.74
***SCGB3A1***_***E55***_***R***	3.05	<2.2E-16	0.80	0.77
***HOXA9***_***E252***_***R***	2.99	<2.2E-16	0.78	0.76
***TFAP2C***_***E260***_***F***	2.98	<2.2E-16	0.81	0.71
***FGF3***_***P171***_***R***	2.96	<2.2E-16	0.78	0.75
***PDGFRB***_***P343***_***F***	2.95	<2.2E-16	0.67	0.55
***NPY***_***P295***_***F***	2.92	<2.2E-16	0.81	0.75
***ASCL2***_***P360***_***F***	2.90	<2.2E-16	0.82	0.70
***LRRC32***_***P865***_***R***	2.90	<2.2E-16	0.81	0.44
***CDK10***_***E74***_***F***	2.88	<2.2E-16	0.83	0.65
***HFE***_***E273***_***R***	2.87	<2.2E-16	0.80	0.75
***SOX1***_***P294***_***F***	2.86	<2.2E-16	0.79	0.70
***TAL1***_***P594***_***F***	2.83	<2.2E-16	0.78	0.73
***RASGRF1***_***E16***_***F***	2.80	<2.2E-16	0.64	0.73
***WT1***_***P853***_***F***	2.79	<2.2E-16	0.78	0.73
***HLF***_***E192***_***F***	2.77	<2.2E-16	0.80	0.75
***GUCY2D***_***E419***_***R***	2.75	<2.2E-16	0.83	0.06
***HS3ST2***_***E145***_***R***	2.75	<2.2E-16	0.84	0.79

^a^ Indicates the adjusted fold change in the β value in the YST compared with the other histologic subtypes of GCT.

^b^ Indicates the change in the β value in the tumor sample compared to the paired normal adjacent in the two YST with available normal tissue.

We selected 5 CpG loci with significant methylation differences by tumor histology (q-value < 2.2E-16 and fold-change > 2.50) for validation by Pyrosequencing (*HOXA9*_E252_R, *SOX1*_P294_F, *WT1*_E32_F, *WNT2*_P217_F, *MDR1*_seq_42_S300_R). Array methylation was significantly correlated with Pyrosequencing methylation for all CpG loci (HOXA9: r = 0.92, p < 0.0001; SOX1: r = 0.92, p < 0.0001; WT1: r = 0.93, p < 0.0001; WNT2: r = 0.97, p < 0.0001; MDR1: r = 0.97, p < 0.0001).

Using an Ingenuity Core Pathway Analysis, the human embryonic stem cell pluripotency (p = 0.02), embryonic stem cell differentiation into cardiac lineages (p = 0.04), serotonin receptor signaling (p = 0.04), and role of Wnt/GSK-3β signaling in the pathogenesis of influenza (p = 0.05) pathways were enriched in CpG loci that had significantly higher methylation in YSTs compared with the other histologic types (q-value < 0.05, fold change > 1.0). Of these, the human embryonic stem cell pathway contains a number of genes that are highly relevant in germ cell biology (*TCF4*, *WNT10B*, *BDNF*, *FGF2*, *BMP3*, *FZD9*, *WNT2*, *APC*, *SOX2*, *NTRK2*, *NTRK3*, *TGFB3*, *TGFB2*, *WNT1*, *PDGFRB*). All of these genes had increased methylation in YST compared to other histologic subtypes, with 9/15 genes showing a greater than 2-fold increase (data not shown).

**Figure 3:**
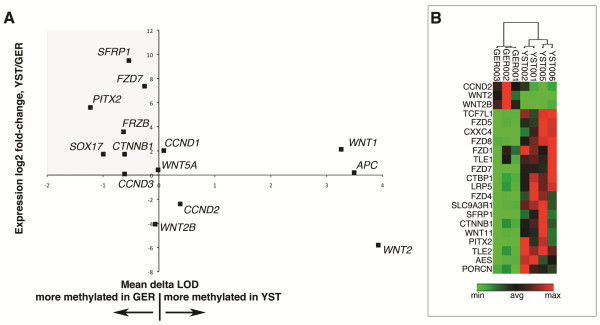
**Correlation of methylation status and expression level for selected Wnt pathway genes. A.** Log_2_fold-change in expression of selected Wnt pathway genes in GER compared to YST plotted as a function of methylation level (expressed as the mean delta LOD). Of the 24 genes profiles, 12 exhibit higher expression and less methylation in YSTs (gray rectangle). **B.** Unsupervised clustering of Wnt pathway gene expression in pediatric gem cell tumors. The genes shown are differentially expressed in germinomas compared to yolk sac tumors (p ≤ 0.05 by two-tailed t-test). Red indicates high expression and green low expression.

To determine if differential methylation of Wnt pathway genes affected the expression of the Wnt pathway in pediatric GCTs, we prepared RNA from fresh-frozen specimens of 7 of the tumors and performed quantitative RT-PCR of selected Wnt pathway genes (15 genes representing 25 methylated loci). Despite the fact that YSTs in general showed higher levels of methylation, of the 15 genes assessed 8 showed both lower levels of methylation and higher expression in YSTs compared to GER (Figure 3A; Additional file 1: Table S1). To further understand the transcriptional landscape of Wnt pathway activation in GCTs, we profiled a total of 84 genes comprising ligands, receptors, effectors and transcriptional targets in the Wnt pathway. Unsupervised clustering based on differential gene expression segregated YSTs and GERs and indicated higher levels of Wnt pathway gene expression in YSTs (Figure 3B; Additonal file 1: Tables S2-S8, Thus the Wnt pathway is active in YSTs and this activity may be explained at least in part by differential methylation.

### Comparison of methylation in normal and tumor samples

Paired normal adjacent tissue was also available for five tumors (2 dysgerminomas, 2 YSTs, and 1 teratoma). While the small sample size limits our ability to perform robust statistical analyses, the correlation coefficient for methylation β values was higher for paired normal/germinoma samples (0.87 and 0.92) and normal/teratoma (0.98) than for paired normal/YST (0.57 and 0.62). Using a change in β (Δβ) > 0.20 to designate a significant difference in methylation between normal and tumor, we found that 425 and 428 CpG loci were differentially methylated in the paired YST samples while 239 and 160 were differentially methylated in the paired dysgerminoma samples and only 15 were differentially methylated in the paired teratoma sample. The Δβ for the paired YST samples was large for the 23 genes that had the largest fold change in the comparison by tumor histology (Δβ for paired samples shown in Table 2), suggesting that methylation at these CpG loci also distinguishes YST from normal testis or ovarian tissue.

### Comparison of mature and immature teratomas

The molecular differences between mature and immature pediatric teratomas have not been explored. When we used RPMM to evaluate methylation differences only among the teratomas, tumor histology was not significantly associated with methylation class (p = 0.11). We also did not see significant differences by sex (p = 0.10), tumor location (p = 0.13) or age (p = 0.28). When we evaluated the individual CpG loci, we identified 190 CpG loci with significant methylation differences after correction for multiple testing. Of these, the majority (96%) had lower methylation in immature teratomas compared with mature teratomas. Using an Ingenuity Core Pathway Analysis, we identified 13 overlapping pathways enriched in CpG loci that had significantly reduced methylation in immature teratomas compared with mature teratomas (Table 3), including a number of pathways related to stem cell biology.

**Table 3:** Significantly enriched pathways with reduced methylation in immature teratomas compared with mature teratomas

**Ingenuity Canonical Pathway**	**Genes**	**p-value**
Role of Oct4 in Mammalian Embryonic Stem Cell Pluripotency	SOX2,CASP6,SPP1,BMI1,RARA,PARP1	0.0015
Axonal Guidance Signaling	GLI2,BMP4,BDNF,BMP2,PIK3R1,EGF,VEGFB,KRAS,LIMK1,PTCH2,EPHB1,GLI3,NGFR,DCC,EFNB3,ERBB2,ITGB1,TUBB3,WNT2B,MMP10,EPHA3,PDGFB,NTRK2,EPHA5,EPHA2	0.0045
Human Embryonic Stem Cell Pluripotency	BMP4,BDNF,BMP2,PIK3R1,FGFR1,WNT2B,TDGF1,FGFR2,PDGFB,APC,SOX2,FGFR3,NTRK2,PDGFRA,CTNNB1,PDGFRB	0.0084
PAK Signaling	ITGB1,MYLK,PIK3R1,PDGFRA,KRAS,EPHA3,TNF,PDGFB,PDGFRB,LIMK1	0.01
PDGF Signaling	ABL2,PIK3R1,MAP3K1,PDGFRA,CAV1,KRAS,EIF2AK2,PDGFB,PDGFRB	0.01
NF-κB Signaling	MAP2K6,BMP4,BMP2,PIK3R1,FGFR1,EGF,FGFR2,KRAS,DDR1,FGFR3,NTRK2,NGFR,KDR,INS,PDGFRA,EIF2AK2,TNF,PDGFRB	0.02
PTEN Signaling	ITGB1,PIK3R1,FGFR1,FGFR2,KRAS,CCND1,DDR1,FGFR3,NTRK2,NGFR,KDR,PDGFRA,PDGFRB	0.03
Transcriptional Regulatory Network in Embryonic Stem Cells	SOX2,ISL1,PAX6	0.03
Estrogen Biosynthesis	CYP2E1,HSD17B12,CYP1B1	0.03
HER-2 Signaling in Breast Cancer	ITGB1,PIK3R1,EGF,KRAS,ERBB3,ERBB2,CCND1,AREG/AREGB	0.04
Gap Junction Signaling	TUBB3,GUCY2D,PIK3R1,CAV1,EGF,KRAS,CTNNB1,HTR2A	0.04
Actin Cytoskeleton Signaling	ITGB1,MYLK,PIK3R1,FGF9,INS,EGF,KRAS,PDGFB,APC,FGF1,LIMK1,MATK	0.04
Embryonic Stem Cell Differentiation into Cardiac Lineages	SOX2,ISL1	0.04

Notably, SOX2 was included in four of the pathways that differed between mature and immature teratomas. We were able to evaluate SOX2 by quantitative RT-PCR in 34 of the samples included in our analysis (N = 17 teratomas). Overall, we found that methylation at SOX2 was negatively correlated with expression (r = -0.40, p = 0.06). We also found that SOX2 expression varied by histologic subtype, with YST and germinoma having lower levels of expression than either group of teratomas, although this difference did not reach statistical significance (p = 0.18, Additonal file 1: Table S9). We also evaluated expression of DNMT3B, a known regulator of de novo methylation. We observed significantly higher levels of DNMT3B expression in YST compared with all other histologic subtypes (p < 0.0001).

### Global LINE1 Methylation

Global methylation at CpG loci in LINE1 elements was measured in a subset of the samples from the CHTN (N=41). We observed significant differences by tumor histology, with both YST (average methylation = 66%, standard deviation (SD) 10%) and dysgerminomas (average methylation = 42%, SD 14%) exhibiting significantly lower methylation levels than normal adjacent (average methylation = 82%, SD 5%), mature teratomas (average methylation = 78%, SD 5%), and immature teratomas (average methylation = 76%, SD 11%) (p < 0.0001). No significant differences in average LINE1 methylation were observed by tumor location (p = 0.39), sex (p = 0.82) or age group (p = 0.36).

### Methylation in imprinted genes

Lastly, methylation in the differentially methylated region (DMR) of imprinted genes differed by tumor histology and location in a subset of the samples (N = 41) (Table 4). The majority of germinomas had lower methylation than expected for an imprinted gene (<33%) at loci that are normally methylated on both the paternal and maternal allele. Methylation patterns in teratomas were dependent on tumor location. In ovarian teratomas, loci that are typically methylated on the paternal allele had reduced methylation in almost all samples while loci that are typically methylated on the maternal allele had increased methylation. In contrast, with the exception of H19 CTCF6, the majority of extragonadal teratomas in both males and females had methylation levels in the normal range for an imprinted locus (33-66%). This was consistent for both mature and immature teratomas (data not shown). The results for YST were more variable, with some samples exhibiting normal methylation levels at all loci while others had either reduced or increased methylation.

**Table 4:** Methylation in imprinted genes by tumor location and histology

	**Females**				**Males**	
**Average Methylation**^**1**^	**YST**	**Germinoma**	**Teratoma**		**YST**	**Teratoma**
			**Ovarian**	**Extragonadal**		
	**N**^2^ **(%)**	**N**^2^ **(%)**	**N**^2^ **(%)**	**N**^2^ **(%)**	**N**^2^ **(%)**	**N**^2^ **(%)**
	**Paternal Allele Methylated**					
***H19 *CTCF3**						
**< 33%**	2 (25)	4 (67)	9 (90)	1 (12)	2 (50)	1 (25)
**33-66%**	6 (75)	2 (33)	1 (10)	7 (88)	1 (25)	3 (75)
**> 66%**	0	0	0	0	1 (25)	0
				0.003		
***H19 *CTCF6**						
**< 33%**	4 (50)	5 (83)	9 (90)	2 (29)	2 (50)	3 (75)
**33-66%**	4 (50)	1 (17)	1 (10)	5 (71)	2 (50)	1 (25)
**> 66%**	0	0	0	0	0	0
				0.04		
***IGF2***						
**< 33%**	2 (29)	4 (67)	8 (80)	1 (12)	0	0
**33-66%**	5 (71)	2 (33)	2 (20)	7 (88)	4 (100)	4 (100)
**> 66%**	0	0	0	0	0	0
				0.02		
	**Maternal Allele Methylated**					
***KvDMR***						
**< 33%**	2 (25)	4 (67)	0	3 (37)	3 (75)	1 (25)
**33-66%**	6 (75)	2 (33)	2 (20)	5 (63)	0	3 (75)
**> 66%**	0	0	8 (80)	0	1 (25)	0
				<0.0001		
***PEG3***						
**< 33%**	0	6 100)	0	1 (12)	0	0
**33-66%**	6 (75)	0	1 (10)	7 (88)	1 (33)	4 (100)
**> 66%**	2 (25)	0	9 (90)	0	2 (67)	0
				<0.0001		
***SNRPN***						
**< 33%**	3 (38)	6 (100)	1 (10)	2 (25)	4 (100)	0
**33-66%**	5 (62)	0	0	6 (75)	0	4 (100)
**> 66%**	0	0	9 (90)	0	0	0
				<0.0001		

^1^Categories represent three methylation states based on the average percent methylation across all CpG loci analyzed in the DMR: <33% (hypomethylation), 33-66% (median methylation), and >66% methylation (hypermethylation).

^2^ N’s do not sum to total due to missing data.

We also compared methylation at imprinted loci in normal and tumor tissue in the 5 samples with adjacent normal DNA (Table 5). With a few exceptions, the normal adjacent tissue exhibited DNA methylation within the expected range (34 – 66% methylation) in samples where the tumor tissues were outside the expected range (0 – 33% or > 66% methylation).

**Table 5:** Average methylation at imprinted genes in five samples with paired normal adjacent tissue

	**Ovarian Germinoma**	**Ovarian Teratoma**	**Ovarian YST**	**Ovarian Germinoma**	**Testicular YST**
	**Age 7**	**Age 21**	**Age 19**	**Age 11**	**Age 1**
	**Average Methylation**^**1**^	**Average Methylation**^**1**^	**Average Methylation**^**1**^	**Average Methylation**^**1**^	**Average Methylation**^**1**^
**H19 CTCF3**					
**Normal**	48%	48%	61%	49%	50%
**Tumor**	13%	8%	63%	14%	26%
					
**H19 CTCF6**					
**Normal**	36%	36%	44%	34%	36%
**Tumor**	10%	6%	39%	10%	26%
					
**IGF2**					
**Normal**	54%	53%	68%	31%	63%
**Tumor**	12%	33%	26%	9%	55%
					
**KvDMR**					
**Normal**	56%	54%	78%	53%	63%
**Tumor**	11%	97%	24%	10%	11%
					
**SNRPN**					
**Normal**	41%	37%	55%	NA^2^	21%
**Tumor**	10%	81%	19%	40%	2%
					
**PEG3**					
**Normal**	34%	39%	54%	NA^2^	37%
**Tumor**	9%	86%	88%	40%	NA^2^

^1^Average percent methylation across all CpG loci analyzed in the DMR.

^2^NA: Sample failed to amplify.

## Discussion

We identified differential methylation by tumor histology in a series of pediatric GCTs, with evidence that YSTs exhibit promoter hypermethylation in a large number of cancer-related genes while germinomas and teratomas do not. These CpG loci were not hypermethylated in the normal adjacent tissue from two patients with YSTs, suggesting that methylation patterns also distinguish yolk sac tumor tissue from normal ovary or testis tissue. Four pathways, most notably a human embryonic stem cell pathway, were over-represented among the CpG loci that were hypermethylated in YSTs. A smaller number of CpG loci exhibited significantly different methylation in a comparison of mature and immature teratomas, however these loci were strikingly enriched for genes associated with embryonic stem cell pluripotency and developmental signaling pathways, such as PTEN, PDGF and NF-κB. In addition, immature teratomas were enriched for differential methylation of genes involved in axonal guidance signaling, reflecting the neuroepithelial character of these tumors. We also saw differences in global methylation at LINE1 elements and in methylation at imprinted loci by tumor location and histology.

Our results are consistent with the few studies to date that have evaluated promoter hypermethylation in pediatric GCT. Promoter hypermethylation has been identified in three tumor suppressor genes (*APC*[6], *RUNX3*[7] and *HIC1*[8]) in a sample of 10 infant testicular YSTs. Furukawa *et al*. [5] found differences in methylation levels in 2 imprinted genes and 17 tumor suppressor genes by tumor histology, with abnormal epigenetic reprogramming occurring in YSTs but not in seminomas or teratomas. In a more recent study, Jeyapalan *et al*. [9] evaluated both global hypomethylation of LINE-1 elements and promoter specific hypermethylation using the Illumina GoldenGate Cancer Methylation Panel in germinomas and YST (this study did not include teratomas). They found evidence for global hypomethylation in both histologic subtypes of GCT, while promoter hypermethylation was identified only in YST. Jeyapalan et al. [9] identified a list of 33 genes that were hypermethylated in more than 80% of YSTs and in <25% of germinomas. Of these 33 genes, all exhibited significantly increased methylation in the YSTs in our series, with 12 included in the list of 23 CpG loci with greater than 2.75 fold increased methylation in YSTs (Table 2). This hypermethylator phenotype in YSTs was previously reported to be associated with increased expression of DNMT3B [9].

Histologic characteristics of GCTs are dependent on the degree of differentiation that has occurred at the time of transformation [27,28]. Cells that do not undergo differentiation following transformation become germinomas. According to one model, neoplastic cells that undergo differentiation become embryonal carcinomas followed by further differentiation into embryonic (teratomas) or extra-embryonic (choriocarcinoma or YSTs) tumors. In addition, the partial erasure of methylation at imprinted genes in pediatric GCTs suggests that they originate from a germ cell at an earlier stage of development than adult TGCTs, which have complete erasure of methylation at imprinted genes [28]. Despite this difference, studies in adult GCTs have identified methylation differences by tumor histology similar to studies in the pediatric age group. Analyses of methylation in selected tumor suppressor genes [29-31] or global methylation profiles [4,32] have identified increased methylation in non-seminomas, including GCTs with a YST component, compared to seminomas. At this time, it is unclear whether these methylation differences by tumor stage are driving tumor potential of the GCT, or if they reflect the stage of normal embryonic development of the germ cell when transformation occurred.

The lack of a difference in promoter methylation in teratomas and dysgerminomas was somewhat surprising, given that dysgerminomas are undifferentiated while teratomas are differentiated. We did, however, observe differences in global methylation of LINE1 elements in teratomas compared with dysgerminomas. Consistent with our findings, studies of methylation using 5^m^C staining [2] and restriction landmark genomic scanning [1] in adult testicular GCTs have reported that seminomas exhibit global hypomethylation while nonseminomas exhibit widespread methylation. The study by Jeyapalan *et al*. [9] did not include teratomas; however, consistent with our findings, the germinomas could not be distinguished from the normal tissue samples included on the array and did not exhibit promoter hypo- or hypermethylation. They did also observe global hypomethylation in LINE-1 elements in both the germinomas and the YSTs, consistent with previous data suggesting that methylation of global repetitive elements does not always correlate with methylation in the promoter region of genes [33].

Previous studies have detected alterations in imprinting in cancer in both adult and pediatric tumors, including GCTs [10-12] Genomic imprinting is an epigenetic phenomenon (driven by methylation) that results in parent-of-origin specific gene expression. Because PGCs erase their inherited imprint and re-establish the correct sex-specific imprint following arrival on the genital ridge, loss of imprinting (LOI) has been proposed as a marker for the stage of PGC development when the tumor arose [34-37]. Several years ago, we evaluated genomic imprinting of H19/IGF2 in 11 informative tumors from this set of pediatric GCTs [38] and found that LOI occurred in pediatric GCTs. These observations were supported by the findings of an additional small study of imprinting in pediatric GCTs [10]. Here, we evaluated methylation in imprinted genes rather than allele specific expression in order to increase the number of samples with informative results, and we expanded the analysis to include three genes that are typically methylated on the maternal allele. We found significant differences in methylation levels at imprinted loci by tumor histology and location. As expected based on a previous analysis of H19/IGF2 methylation in adult and pediatric GCTs [11], the germinomas exhibited hypomethylation at all loci. Methylation at imprinted loci also distinguished teratomas located in the ovary vs. extragonadal locations, with ovarian teratomas exhibiting hypomethylation at CpG loci typically methylated on the paternal allele and hypermethylation at CpG loci typically methylated on the maternal allele. This finding provides further evidence that ovarian teratomas are parthenogenetic in origin [15,39,40]. Overall, these data provide direct support for the theory that methylation status at imprinted loci in GCTs represents the origin and stage of development of the PGC when transformation occurred [28].

This study population represents a well-characterized sample of pediatric GCTs including samples from all three major histologic subgroups; however, several limitations must be considered when interpreting the results. The number of germinomas was relatively small and consisted only of samples from females, which may have limited our ability to detect differences in methylation in this group. The lack of age-matched normal germ cell tissue limited our ability to detect methylation differences between GCTs and normal germ cells. We also evaluated methylation only in the CpG loci of a relatively small number of genes that have previously been implicated in cancer. A more unbiased evaluation of genome wide methylation would provide a more comprehensive picture of methylation patterns in GCTs.

## Conclusion

These data demonstrate a distinct methylation pattern in YSTs compared to germinomas and teratomas, consisting of hypermethylation at a large number of genes known to be involved in tumorigenesis. The CpG loci identified as hypermethylated in YSTs included in our study overlapped remarkably with the CpG loci identified in two independent series of YSTs recently reported by Jeyapalan *et al*. [9]. Whether these alterations result from exposure to environmental agents *in utero* or simply are a result of abnormal PGC development remains to be elucidated. Further analyses will be required to better understand the functional and therapeutic consequences of this altered methylation signature.

## Abbreviations

GCT: Germ cell tumor; GER: Germinoma; YST: Yolk sac tumor; IT: Immature teratoma; RPMM: Recursively partitioned mixture model; LINE1: Long interspersed noncoding element 1.

## Competing interests

The authors declare that they have no competing interests.

## Authors' contributions

JA conceived of the study and participated in its design and coordination, examined tumor specimens, analyzed data and wrote the manuscript. JAR conceived of the study and participated in its design and coordination. BC analyzed tumor methylation data and application of the RPMM. NJF and KSC examined tumor specimens, prepared samples for analysis and analyzed data. AJH, HN and JKK performed methylation array analysis. DR and ALF provided expert consultation on tumor histology and helped to draft the manuscript. JNP conceived of the study and participated in its design and coordination, examined tumor specimens, analyzed data and wrote the manuscript. All authors read and approved the final manuscript.

## Pre-publication history

The pre-publication history for this paper can be accessed here:

http://www.biomedcentral.com/1471-2407/13/313/prepub

## Supplementary Material

Additional file 1: Table S1 Comparison of Methylation and gene expression. **Table S2.** Wnt pathway gene list. **Table S3.** Average Ct. **Table S4.** Average Delta(Ct). **Table S5.** 2^(-Avg.(Delta(Ct)). **Table S6.** Fold Change. **Table S7.** p-value. **Table S8.** Fold-Regulation. **Table S9.** Expression of SOX2 and DNMT3B by tumor histology. Click here for file
